# Identification of the Chemokine CX_3_CL1 as a New Regulator of Malignant Cell Proliferation in Epithelial Ovarian Cancer

**DOI:** 10.1371/journal.pone.0021546

**Published:** 2011-07-07

**Authors:** Françoise Gaudin, Salam Nasreddine, Anne-Claire Donnadieu, Dominique Emilie, Christophe Combadière, Sophie Prévot, Véronique Machelon, Karl Balabanian

**Affiliations:** 1 UMR_S996, INSERM/Université Paris-Sud 11, Clamart, France; 2 Service de Microbiologie-Immunologie Biologique, Assistance Publique-Hôpitaux de Paris/Hôpital Antoine-Béclère, Clamart, France; 3 UMR_S945, INSERM/Université Pierre et Marie Curie, Assistance Publique-Hôpitaux de Paris/Hôpital de la Pitié-Salpêtrière, Paris, France; 4 Service d'Anatomie et de Cytologie Pathologiques, Assistance Publique-Hôpitaux de Paris/Hôpital Antoine-Béclère, Clamart, France; University of Pennsylvania, United States of America

## Abstract

**Background:**

Little is known about the molecules that contribute to the growth of epithelial ovarian carcinomas (EOC), which remain the most lethal gynecological cancer in women. The chemokine Fractalkine/CX_3_CL1 has been widely reported to play a biologically relevant role in tumor growth and spread. We report here the first investigation of the expression and role of CX_3_CL1 in EOC.

**Results:**

Epithelial cells from the surface of the ovary and the Fallopian tubes and from benign, borderline and malignant tumors all stained positive for CX_3_CL1. In tumor specimens from 54 women who underwent surgical treatment for EOC diagnosis, CX_3_CL1 immunoreactivity was unevenly distributed in epithelial tumor cells, and ranged from strong (33%) to absent (17%). This uneven distribution of CX_3_CL1 did not reflect the morphological heterogeneity of EOC. It was positively correlated with the proliferation index Ki-67 and with GILZ (glucocorticoid-induced leucine zipper), previously identified as an activator of the proliferation of malignant EOC cells. Hierarchical clustering analysis, including age at diagnosis, tumor grade, FIGO stage, Ki-67 index, CX_3_CL1, SDF-1/CXCL12 and GILZ immunostaining scores, distinguished two major clusters corresponding to low and high levels of proliferation and differing in terms of GILZ and CX_3_CL1 expression. *GILZ* overexpression in the carcinoma-derived BG1 cell line resulted in parallel changes in CX_3_CL1 products. Conversely, CX_3_CL1 promoted through its binding to CX_3_CR1 AKT activation and proliferation in BG1 cells. In a mouse subcutaneous xenograft model, the overexpression of *GILZ* was associated with higher expression of CX_3_CL1 and faster tumor growth.

**Conclusion:**

Our findings highlight the previously unappreciated constitutive expression of CX_3_CL1 preceding tumorigenesis in ovarian epithelial cells. Together with GILZ, this chemokine emerges as a regulator of cell proliferation, which may be of potential clinical relevance for the selection of the most appropriate treatment for EOC patients.

## Introduction

Epithelial ovarian cancer (EOC) constitutes the sixth most common cancer and the fifth leading cause of cancer-related death among women in developed countries [1]. Due to the silent nature of early-stage disease, most women with EOC have disseminated disease (*i.e.* expansion in the peritoneum and metastasis in the omentum) at the time of diagnosis and present advanced disease, with a five-year survival rate below 30% [2]. Despite the high incidence and mortality rates of EOC, the etiological factors involved in ovarian carcinogenesis remain poorly defined, limiting the efficacy of treatment protocols.

The epithelial tumor microenvironment consists of a complex tissue containing several cell types. Most of these cells produce and/or respond to chemokines, which may play key roles in the development and progression of primary epithelial tumors [3]–[5]. We have shown, for example, that the α-CXC chemokine Stromal cell-Derived Factor-1 SDF-1/CXCL12 contributes to the immunosuppressive network within the tumor microenvironment, notably by orchestrating the recruitment of pre-DC2s [6]. We have also shown that CXCL12 regulates tumor angiogenesis and that this is critical for tumor growth [7]. By contrast, little if anything is known about the role of the chemokine Fractalkine/CX_3_CL1 in EOC, although it has been evidenced to mediate strong cell adhesion [8] and its presence in epithelial tissues is widely documented [9]–[10]. CX_3_CL1 exists in two forms. The membrane-anchored form mediates the firm adhesion of cells expressing its sole receptor, CX_3_CR1, to the endothelium under physiological flow, through its own intrinsic adhesion function and through integrin activation [11]–[12]. The soluble form is released through cleavage at a site close to the membrane [13]. Like other conventional chemokines, it recruits immune cells bearing CX_3_CR1, such as T lymphocytes and cytotoxic NK cells, dendritic cells or a large subpopulation of CD14^+^ monocytes [8]. As a result of both the adhesion and chemoattractant activities of the chemokine, the CX_3_CL1/CX_3_CR1 complex may mediate either pro- or anti-tumor effects [14]. Pancreatic ductal adenocarcinoma cells bearing CX_3_CR1 specifically adhere to CX_3_CL1-expressing cells of neural origin and migrate in response to CX_3_CL1 produced by neurons and nerve fibers, contributing to perineural dissemination in pancreatic cancer [15]. Prostate cancer cells that express CX_3_CR1 adhere to human bone marrow endothelial cells and migrate toward a medium conditioned by osteoblasts, which secrete the soluble form of the chemokine contributing to the high likelihood of prostate cancer cells metastasizing to the skeleton [16]–[17]. By contrast, soluble CX_3_CL1 (sCX_3_CL1) released in the tumor microenvironment may be an active component of the anti-tumor response [18]–[21], making the vaccination of mice with carcinoma cells modified to produce CX_3_CL1 a potent anti-tumor response due to the chemoattraction of NK cells [22], or making CX_3_CL1 expression by colon cancer cells a factor that drastically reduced their metastatic potential [23].

In the present work, we have investigated the expression of CX_3_CL1 in healthy and malignant ovarian tissues and its role in the proliferation of malignant ovarian epithelial cells. This chemokine was produced by both healthy and malignant ovarian epithelial cells, and its production in EOC was positively correlated to cell proliferation index. Interestingly, it was also correlated to the expression of glucocorticoid-induced leucine zipper (GILZ), a 17 kDa leucine zipper protein discovered as a dexamethasone-induced transcript in murine thymocytes, and that we have recently shown to enhance cell proliferation in EOC and to activate AKT, a crucial signaling molecule in tumorigenesis [24], [25]. Our findings were supported by parallel and complementary data accumulated in tumor specimens from patients diagnosed for EOC, in the BG-1 ovarian cancer cell line and in a mouse subcutaneous xenograft model. They provide further insight into the role of CX_3_CL1 in malignant cell proliferation and tumor growth, closely associated with GILZ.

## Results

### Detection of CX_3_CL1 in healthy and malignant ovarian tissues

**Figure 1:**
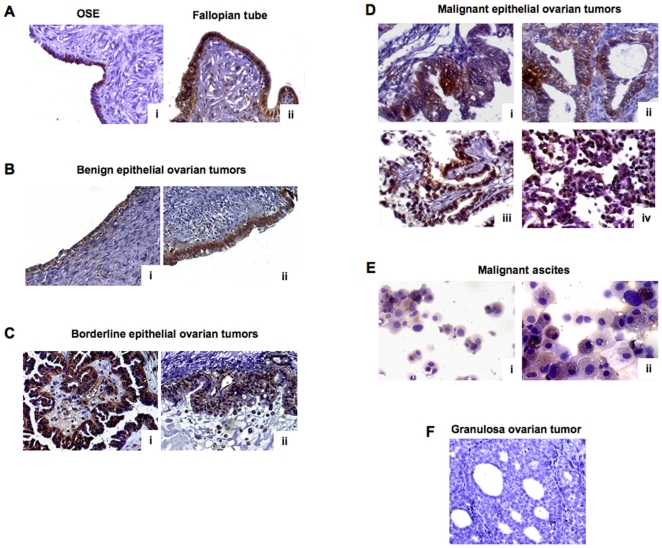
CX_3_CL1 immunoreactivity in healthy and malignant ovaries. (A) healthy ovary, CX_3_CL1 immunoreactivity in the OSE (i) and Fallopian tube (ii). (B) Serous (i) and mucinous (ii) benign ovarian epithelial tumors. (C) Serous (i) and mucinous (ii) borderline ovarian epithelial tumors. (D) Malignant epithelial ovarian tumors: mucinous (i), endometrioid (ii), clear-cell (iii) and serous (iv), CX_3_CL1 immunoreactivity in epithelial cells is confined to the cytoplasm, no staining in the nuclei of tumor cells. (E) Cytocentrifuged CD326^−^ non epithelial (i) and CD326^+^ epithelial (ii) cells isolated from malignant ascites collected from a patient diagnosed with invasive EOC. CX_3_CL1 is detected in CD326^+^ cells and also in some CD326^−^ cells. (F) Non-epithelial ovarian granulosa cell tumor, absence of CX_3_CL1 immunostaining. (A–F) Magnification x 40.

The cellular expression of CX_3_CL1 was examined by immunohistochemisty (IHC) in sections isolated from three healthy ovaries, eight serous and mucinous benign tumors (some still containing normal ovarian tissue), eight serous and mucinous borderline tumors, two ovarian granulosa cell tumors, and from 54 specimens of invasive EOC. CX_3_CL1 was clearly detected in the ovary surface epithelium (OSE) cells and in the epithelium of the Fallopian tubes (Figure 1A). In serous and mucinous benign and borderline tumors, CX_3_CL1 immunoreactivity was detected in proliferating tumor cells derived from the epithelium (Figure 1, B and C). CX_3_CL1 expression was detected in EOC specimens, including the serous, clear-cell, endometrioid and mucinous histological subtypes (Figure 1D). In these tumors, it was detected mostly in malignant cells, making tumor cells the most significant source of CX_3_CL1 in EOC. Consistent with this finding, CX_3_CL1 was detected in epithelial cells from malignant ascites, with higher levels of expression in the CD326^+^ fraction (Figure 1E). CX_3_CL1 was confined to the cytoplasm of malignant epithelial cells and was not detected in nuclei. CX_3_CL1 was absent from non-epithelial ovarian granulosa cell tumors (Figure 1F).

**Figure 2:**
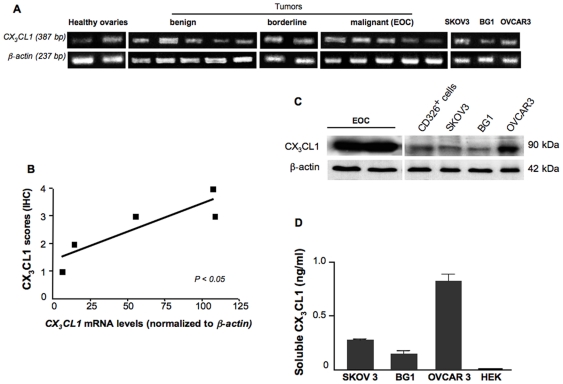
Steady-state levels of CX_3_CL1 products in healthy and malignant ovaries. (A) *CX_3_CL1* mRNA was detected by conventional PCR at the expected size (387 bp) in representative specimens from 2 healthy ovaries, 5 cystadenomas (benign tumors), 2 borderline tumors, 5 adenocarcinomas (malignant tumors) and in the EOC-derived cell lines, SKOV3, OVCAR3 and BG1. The white vertical line separates lanes not run on the same gel. (B) *CX_3_CL1* mRNA levels were quantified by real-time PCR and are expressed as *CX_3_CL1* content normalized to that of *ß-actin*. The diagram shows the distribution of immunostaining scores versus the amount of *CX_3_CL1* mRNAs normalized to those of *ß-actin* for 5 EOC samples. Each symbol represents an individual sample run in triplicate (mean value); Spearman's test, *P*<0.05, r = 0.88. (C) CX_3_CL1 immunoblots of total protein lysates from EOC specimens, from CD326^+^ epithelial cell-enriched malignant ascites samples, and from SKOV3, BG1 and OVCAR3 cell lines. The CX_3_CL1 protein is indicated as a ∼90 kDa band. ß-actin levels are shown for normalization. (D) Detection by ELISA of sCX_3_CL1 in the 24 h culture medium of BG1, OVCAR3 and SKOV3 cells (data are means ± SEM of three separate experiments); undetectable sCX_3_CL1 in the culture medium of HEK-293T cells (HEK), used as a negative control.

Messenger RNA for CX_3_CL1 was visualized by RT-PCR, which generated a product of the expected size (387 bp) from three healthy ovary samples, in six specimens from benign ovarian tumors, three borderline specimens and nine EOC specimens. It was also detected in the BG1, SKOV3 and OVCAR3 ovarian cancer cell lines. Representative data are shown in Figure 2A. The amount of mRNA was quantified by real time PCR on five EOC specimens. It was positively correlated with the intensity of IHC staining on a seven-point scale (Spearman's test, *P*<0.05, r = 0.88) (Figure 2B). A 90-kDa protein, corresponding to the expected size of the full-length CX_3_CL1, was detected in EOC biopsy samples, in CD326^+^ epithelial cells from malignant ascites and in SKOV3, BG1 and OVCAR3 cells (Figure 2C). CX_3_CL1 is a membrane-bound molecule with the chemokine domain on a mucin-like stalk. Cleavage at the base of this stalk by metalloproteinases generates a soluble chemokine, which functions as a classical chemoattractant [13]. We then investigated whether sCX_3_CL1 was released from ovarian cancer cells. It was detected in malignant ascites (ranging from 1.3 to 1.5 ng/ml). We also carried out ELISA assays on culture supernatants. The largest amounts of sCX_3_CL1 were recovered from the culture supernatant of OVCAR3 cells, which gave the strongest signal on western blots (Figure 2D). Our findings highlight the previously unappreciated constitutive expression of CX_3_CL1 on healthy epithelia of the ovary surface and Fallopian tubes, indicating that EOC may originate from either of these epithelia. We further reveal that CX_3_CL1 production by malignant epithelial cells precedes tumorigenesis.

### CX_3_CL1 is correlated with Ki-67 and GILZ in EOC

**Figure 3:**
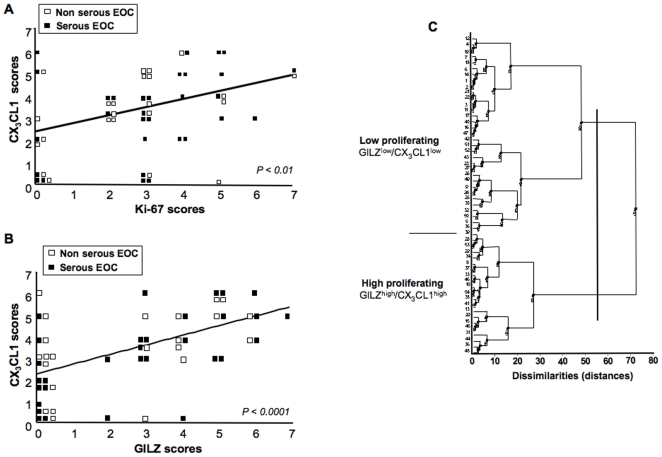
Correlation of CX_3_CL1 and Ki-67 and GILZ in EOC. (A and B) CX_3_CL1 and Ki-67 final scores (A, Spearman test, *P*<0.01, r = 0.38) and CX_3_CL1 and GILZ final scores (B, Spearman test, *P*<0.0001, r = 0.59) were positively correlated in 54 EOC specimens including serous (black squares) and non serous (white squares) samples. (C) Dendrogram generated by hierarchical agglomerative cluster analysis for the 54 EOC specimens studied, against age at diagnosis, FIGO stage, grade, Ki-67, GILZ, CX_3_CL1 and CXCL12 immunoreactivity levels. Two clusters are identified, with low (top) and high (bottom) levels of proliferation. Relevant specimens are labeled with numbers.

CX_3_CL1 immunostaining was heterogeneous in EOC specimens, spanning from the absence of detectable staining (score 0, 9/54) to strong immunoreactivity (scores 5-7, 18/54). We therefore investigated whether differences in CX_3_CL1 expression levels were associated with the expression of two markers of proliferation: Ki-67, which is routinely used for diagnosis [26] and GILZ, which we recently identified as a factor controlling the proliferation of malignant EOC cells [25]. Immunoreactivity for CX_3_CL1, GILZ and Ki-67 was scored on a seven-point scale on the basis of staining intensity and the degree of staining of serial sections of fragments of EOC from 54 patients. There was a highly significant positive correlation between the scores for Ki-67 and those for GILZ, as expected (Table 1). Interestingly, significant positive correlations were found between CX_3_CL1 and Ki-67 and between CX_3_CL1 and GILZ, for the entire cohort of 54 patients (Figure 3, A and B). The immunostaining scores for these proteins were also correlated in serous carcinoma and non serous carcinoma (Table 1).

**Table 1:** Correlations of CX_3_CL1, Ki-67 and GILZ immunoreactivity in EOC specimens.

	CX_3_CL1 / Ki-67	CX_3_CL1 / GILZ	GILZ / Ki-67
All EOC specimens	ar = 0.38	ar = 0.59	ar = 0.57
N = 54	a *P*<0.005	a *P*<0.00001	a *P*<0.00001
Serous specimens	ar = 0.42	ar = 0.64	ar = 0.57
N = 30	a *P*<0.05	aP<0.0001	a *P*<0.001
Non serous specimens	ar = 0.45	ar = 0.57	ar = 0.57
N = 24	a *P*<0.05	a *P*<0.005	a *P*<0.005

a Spearman's test.

CXCL12, a chemokine produced by ovarian cancer cells [6], has been implicated in the control of proliferation in these cells [27]. As we have previously shown in a cohort of 183 patients [28], CXCL12 immunoreactivity in cancer cells was heterogeneous, with scores of 0 (undetectable production) obtained in 16 patients and of 5 to 7 (strong immunoreactivity) obtained in eight patients in our cohort of 54 patients. There was no significant correlation between final scores for CXCL12 and CX_3_CL1, or between final scores for CXCL12, GILZ and Ki-67. An analysis of seven datasets, including age at diagnosis, FIGO stage, grading, GILZ, Ki-67, CX_3_CL1 and CXCL12 immunoreactivities, based on an agglomerative hierarchical clustering approach revealed two major clusters, as shown by the dendrogram generated from the statistical analysis (Figure 3C). The main characteristics distinguishing the two major clusters, corresponding to low and high levels of proliferation, are presented in Table 2. As expected, CXCL12 production did not differ significantly between the two groups. By contrast, CX_3_CL1 and GILZ immunoreactivities in tumor cells were higher for the group with the higher level of proliferation. Despite the relatively small number of patients, the number of cancers at FIGO stages III and IV was significantly higher in the high proliferation group. By contrast, age at diagnosis and grade did not differ between the two groups. Thus, higher rates of proliferation were associated with the upregulation of GILZ and CX_3_CL1 expression.

**Table 2:** Identification of high- and low-proliferation clusters.

			Fisher's test
			statistical
	Patient distribution		significance
	Cluster 1	Cluster 2	
	Low proliferating	High proliferating	
All carcinomas	33	21	
Age at diagnosis			
<60 year	21	10	
>60 year	12	11	NS
Histological types			
Serous	17	13	
Non serous	16	8	NS
Clear cells	6	0	*P*<0.05
Mucinous	5	3	NS
Endometrioid	4	3	NS
Undifferentiated	1	2	NS
Figo stages			
IA-IIC	18	1	
IIIA-IV	15	20	*P*<0.0001
Grades			
≤2	18	12	
>2	15	9	NS
Ki-67 Immunostaining			
Low scores (0–3)	29	7	
High scores (4–7)	4	14	*P*<0.0001
GILZ immunostaining			
Low scores (0–3)	27	6	
High scores (4–7)	6	15	*P*<0.0001
CX_3_CL1 immunostaining			
Low scores (0–4)	27	10	
High scores (5–7)	6	11	*P*<0.01
CXCL12 immunostaining			
Low scores (0–2)	16	12	
High scores (3–7)	17	9	NS

### GILZ upregulates CX_3_CL1 expression

**Figure 4:**
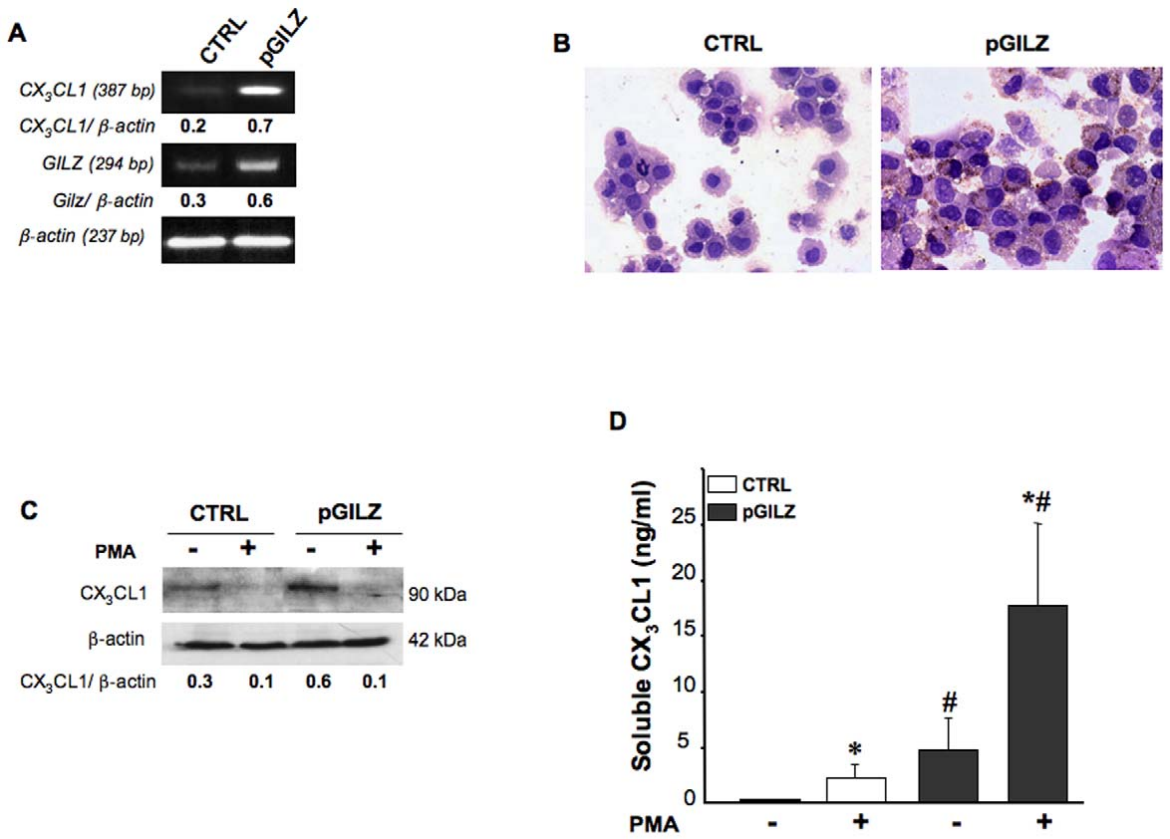
*GILZ* upregulation increases CX_3_CL1 levels in ovarian epithelial malignant cells. (A) *CX_3_CL1* PCR signal intensity in CTRL and pGILZ BG-1 cells was quantified by densitometry with normalization against the signal for *ß-actin*; results are expressed as *CX_3_CL1/ß-actin* ratios. One experiment representative out of three. (B) Immunostaining for CX_3_CL1 on CTRL and pGILZ BG1 cytocentrifuged cells. Magnification x 40. (C) Total cellular protein extracts of CTRL and pGILZ BG1 cells cultured with or without 100 ng/ml PMA for 24 h were analyzed by western blotting with a specific Ab recognizing the extracellular domain of CX_3_CL1. CX_3_CL1 levels were quantified by densitometry, with normalization against the signal for ß-actin; results are expressed as CX_3_CL1/ß-actin ratios. One experiment representative of three. (D) Histograms show the release of sCX_3_CL1 into the supernatant of cells treated or not with PMA, as measured by ELISA. Results are the means ± SEM of three independent experiments. *P<0.05, absence versus presence of PMA and ^#^ *P<0.05*, CTRL versus pGILZ BG1 cells (unpaired *t* test).

Immunohistochemical data clearly indicated that GILZ levels in malignant cells were positively correlated with CX_3_CL1 levels, suggesting a possible role for GILZ in regulating CX_3_CL1 production. We tested this hypothesis by determining the amounts of CX_3_CL1 mRNA and protein in pGILZ (overexpressing GILZ) and CTRL (producing low amount of GILZ) BG1 cells. As expected, GILZ content (mRNA and protein) was significantly higher in pGILZ than in CTRL clones. Parallel increases in CX_3_CL1 mRNA and protein were depicted on RT-PCR, IHC and western blots (Figure 4, A, B and C). In addition, the pGILZ cells released larger amounts of sCX_3_CL1 than the control cells (Figure 4D). Using an Ab targeting the extracellular domain of CX_3_CL1, western blots of lysates of BG1 cells treated with phorbol-12-myristate-13-acetate (PMA), a protein kinase C (PKC) activator, showed an absence of the 90-kDa band corresponding to the full-length of CX_3_CL1. Conversely, this treatment increased the release of sCX_3_CL1 into the supernatant. (Figure 4, C and D). Taken together, our results indicate that CX_3_CL1 production by ovarian epithelial malignant cells is upregulated by GILZ.

### CX_3_CL1 increases malignant cell proliferation

**Figure 5:**
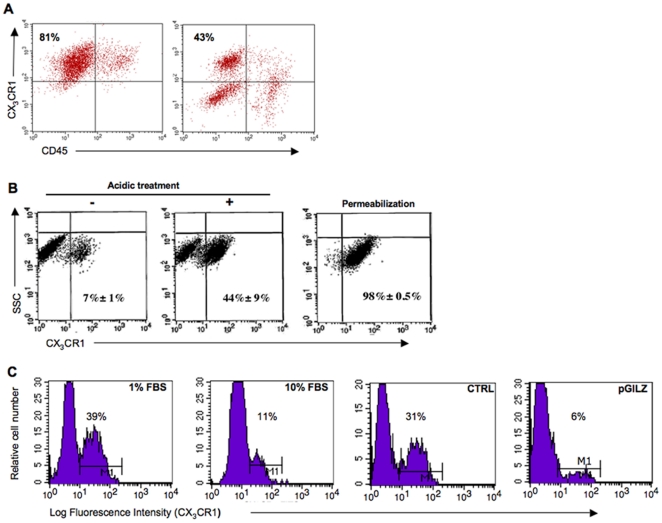
Expression of CX_3_CR1 in ovarian epithelial malignant cells. (A) Levels of CX_3_CR1 and of the pan-hematopoietic marker CD45 were determined by flow cytometry in 2 freshly dissociated samples of EOC specimens. Numbers indicate frequencies of CX_3_CR1^+^ CD45^−^ cells. (B) Representative FACS profiles for CX_3_CR1 levels in BG1 cells. Left, surface expression of CX_3_CR1 under basal conditions; middle, surface expression of CX_3_CR1 in cells after acidic treatment; right, expression of CX_3_CR1 in permeabilized cells. Numbers indicate percentage of CX_3_CR1^+^ cells (mean ± SEM) for 3 independent experiments. (C) Histograms show the fluorescence intensity of CX_3_CR1 staining at the surface of CTRL BG1 cells cultured in the presence of 1% or 10% FBS, and of CTRL and pGILZ BG1 cells cultured in the presence of 1% FBS. Numbers indicate the percentage of CX_3_CR1^+^ cells for one representative experiment out of three.

**Figure 6:**
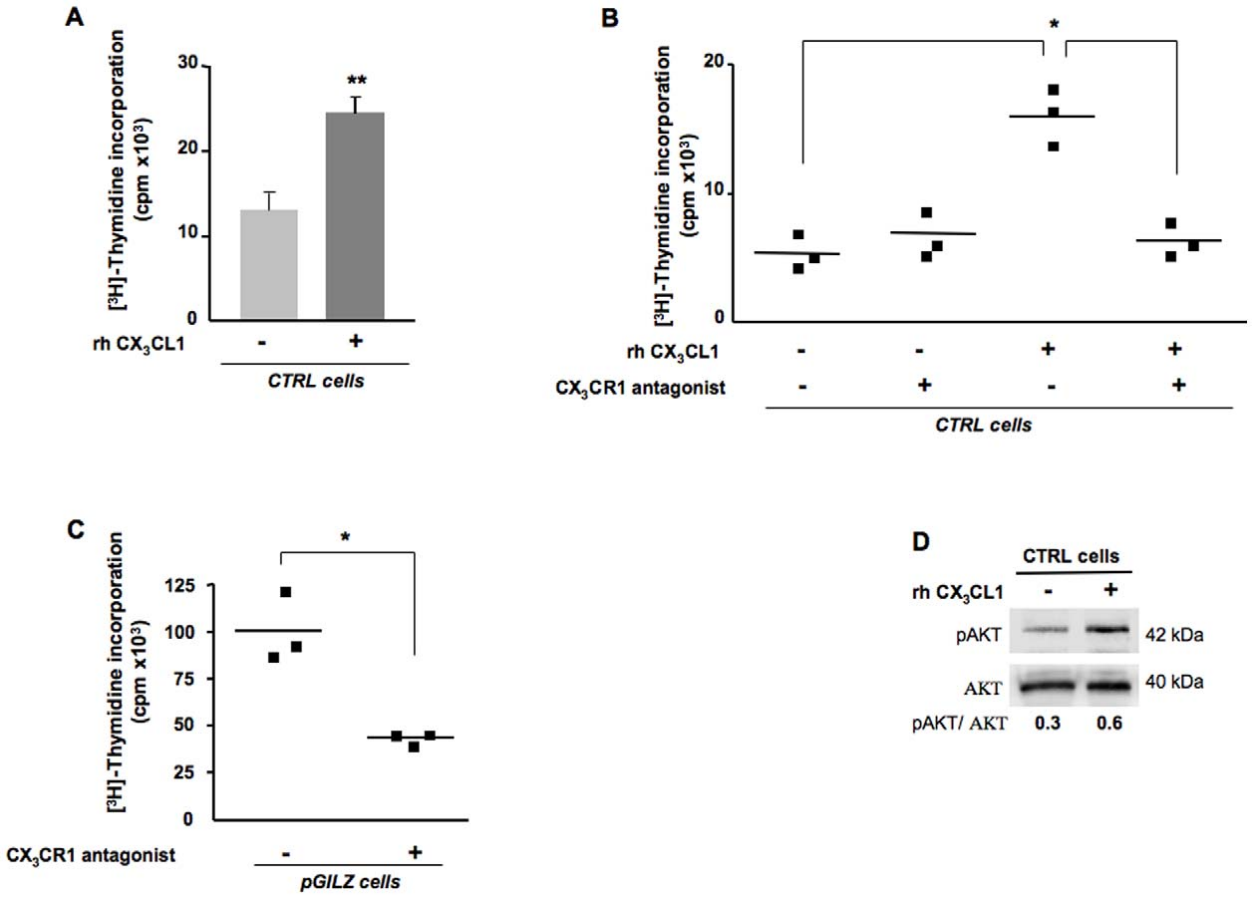
CX_3_CL1 promotes the proliferation of BG1 cells. (A–C) Proliferation was measured by [^3^H]-thymidine incorporation (A) in CTRL cells incubated with or without 10 ng/ml rhCX_3_CL1 for 24 h, 9 independent experiments. Histograms represent means ± SEM, paired t test, ^**^ *P*<0.001; (B) in CTRL cells incubated with or without 10 ng/ml rhCX_3_CL1 for 24 h, in the presence or absence of 10 µg/ml CX_3_CR1 antagonist; each symbol represents an individual sample run in triplicate, lines represent mean values, * *P*<0.05, *t* test, one representative experiment out of 3; (C) in pGILZ cells with and without treatment with 10 µg/ml CX_3_CR1 antagonist replaced every 24 h for 72 h, each symbol represents an individual sample run in triplicates, lines represent mean values, * *P*<0.05, *t* test, one representative experiment out of 3. (D) Total cellular protein extracts of CTRL cells cultured in the presence and absence of 10 ng/ml rhCX_3_CL1 analyzed by western blotting with specific Abs. pAKT levels were quantified by densitometry, with normalization against the signal for total AKT. Results are expressed as pAKT/AKT ratios. One blot, representative of three carried out, is shown.

We showed that sCX_3_CL1 is released by ovarian cancer cells from the shedding of the membrane-bound chemokine suggesting that sCX_3_CL1 may be an active component of the tumoral microenvironment. Here, we asked whether this chemokine has an impact on tumor cell proliferation, as suggested by the correlation of CX_3_CL1 and Ki-67 immunostainings in EOC specimens. This proliferative action may result from an autocrine effect of CX_3_CL1, which depends on the expression of its unique receptor, CX_3_CR1 [8]. We detected *CX_3_CR1* mRNA by conventional RT-PCR at the expected size (340 bp) in all the EOC specimens tested (N = 14) and in the three EOC cell lines, BG1, SKOV3 and OVCAR3. Flow-cytometric analyses further revealed membrane CX_3_CR1 expression in both CD45^+^ and CD45^−^ cells from malignant tumor specimens. In the CD45^−^ fraction, which is highly enriched in malignant epithelial ovarian cells, the percentage of CX_3_CR1^+^ cells ranged from 20% to 95% (Figure 5A). In BG1 cells, the steady-state level of membrane CX_3_CR1 expression was weak (<10% of total cells) under basal conditions (Figure 5B). Interestingly, the fraction of CX_3_CR1^+^ cells was markedly increased by acidic treatment, a process known to dissociate the ligand from its receptor. On another side, decreasing the concentration of FBS from 10% to 1% in culture medium led to increased membrane expression of CX_3_CR1 in BG1 cells (Figure 5C). In contrast, the level of surface CX_3_CR1 was lower in pGILZ cells, which produce higher amounts of sCX_3_CL1 than CTRL cells. Based on these findings, we used CTRL BG1 cells cultured in medium supplemented with 1% FBS to measure the proliferative effect of recombinant human (rh)CX_3_CL1 by [^3^H]-thymidine incorporation. Results from nine independent experiments showed that rhCX_3_CL1 roughly doubled the rate of cell proliferation after 24 h treatment (Figure 6A). This response was abrogated by the addition of a CX_3_CL1 analog with a modified N-terminus that binds to CX_3_CR1 and acts as an antagonist (Figure 6B) [29]. These results show a proliferative action of exogenous CX_3_CL1 through its binding to CX_3_CR1. We next investigated whether endogenous CX_3_CL1 stimulates tumor cell proliferation. For this purpose, pGILZ BG1 cells, which generate high amounts of endogenous CX_3_CL1, were treated with the CX_3_CL1 analog renewed every 24 h for 72 h. Under these conditions, the rate of cell proliferation was markedly decreased, underlying a role for endogenous CX_3_CL1 in regulating tumor cell proliferation (Figure 6C).

AKT hyperactivation is frequently observed in ovarian cancers and is related to the control of cell proliferation in EOC [30], [31] [32]–[33]. Levels of pAKT, which is the active AKT form, were higher in rhCX_3_CL1-treated BG1 cells (Figure 6D). These results strongly suggest that the proliferative effect of the CX_3_CL1/CX_3_CR1 couple is associated with AKT activation. GILZ has been previously identified as a proliferative factor activating AKT in EOC [25]. To confirm that CX_3_CL1 action involves AKT activation *in situ*, we measured Ki-67 and pAKT scores on EOC specimens scored 0 for GILZ and either producing or not CX_3_CL1. As shown in Table 3, proliferation and AKT phosphorylation were higher in specimens producing CX_3_CL1. Altogether, these findings suggest that the proliferative effect of CX_3_CL1 on ovarian epithelial malignant cells is consecutive to CX_3_CR1 binding that activates AKT.

**Table 3:** Impact of CX_3_CL1 on cell proliferation and pAKT content in GILZ-negative EOC specimens (scored 0).

	CX_3_CL1 (scored 0)	CX_3_CL1 (scored 2–6)	Student *t* test
	N = 6	N = 15	
Ki-67 scores (mean ± SEM)	0. 5±0.5	2±0.4	*P*<0.05
% pAKT high	20%	64.2%	

### *In vivo* impact of GILZ overexpression

**Figure 7:**
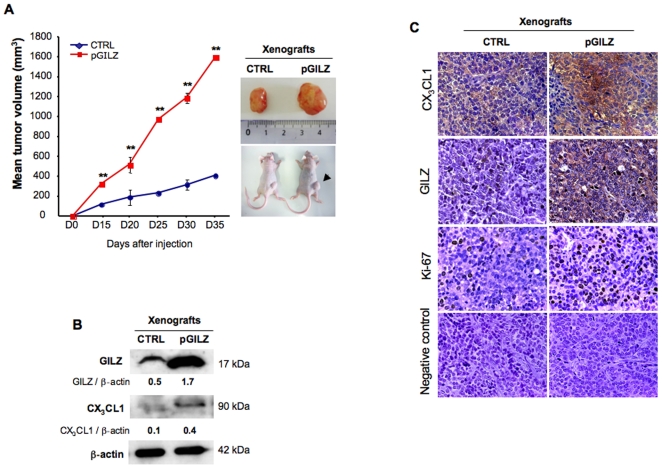
Impact of *GILZ* overexpression in xenografted tumors. (A) pGILZ or CTRL BG1 cells (40×10^6^ cells/ml) were injected subcutaneously into the right flanks of nude mice. Tumor size was measured every 5 days, for 35 days (N = 3 mice per group). Tumor volume [mm^3^] was calculated as follows: (length [mm]) × (width [mm])^2^×0.5.** *P*<0.001 (unpaired *t* test). (B) Total cellular protein extracts of xenografted tumors were analyzed by western blotting with specific Abs. CX_3_CL1 and GILZ levels were quantified by densitometry, with normalization against the signal for ß-actin; results are expressed as CX_3_CL1 or GILZ/ß-actin ratios. One blot representative of three carried out is shown. (C) Serial sections of pGILZ and CTRL xenografted tumors were stained for CX_3_CL1, GILZ and Ki-67. Negative control: no labeling was detected when each primary Ab was omitted. Magnification x 40.

Finally, we investigated the impact of GILZ and CX_3_CL1 on tumor growth *in vivo* by using a mouse subcutaneous xenograft model. BG1 cells either overexpressing GILZ (pGILZ) or not (CTRL) were injected subcutaneously into athymic nude mice and tumor growth was followed for 35 days. The mean tumor volumes are represented graphically in Figure 7A. The tumors developing from pGILZ cells had significantly larger volumes than those developing from CTRL, at any given time point. Western blots of xenograft extracts and immunostaining showed parallel increases in GILZ and CX_3_CL1 protein levels (Figure 7, B and C). Thus, *GILZ* overexpression is clearly associated with higher levels of CX_3_CL1 production in tumors, resulting in higher rates of proliferation and tumor growth.

## Discussion

In this study, we unveiled that CX_3_CL1 was constitutively produced in EOC and investigated the role of this chemokine in tumor growth. The production of this chemokine preceded malignancy in the OSE, and was also found in the Fallopian tubes of healthy women and in benign tumors. Immunohistological analysis revealed that CX_3_CL1 production in EOC samples was correlated with levels of Ki-67 and GILZ, two markers of proliferation in malignant ovarian epithelial cells. Hierarchical clustering analysis identified two major clusters, with high and low levels of proliferation, differing in GILZ and CX_3_CL1 levels. *In vitro*, GILZ overproduction leads to an increase in CX_3_CL1 production in BG1 cells. CX_3_CL1 increases BG1 cell proliferation via its receptor, CX_3_CR1, and a parallel increase is observed in pAKT levels. In xenografted mice, the overexpression of both GILZ and CX_3_CL1 is associated with faster tumor growth. These results highlight a relationship between GILZ and CX3CL1 as a key regulator of malignant cell proliferation and tumor growth.

According to recent hypotheses concerning the origin and histogenesis of EOC, type I tumors, which are believed to include all major histotypes, originate from the OSE, which was traditionally considered to be the source of the neoplastic transformation. By contrast, type II tumors, which are thought to comprise almost exclusively high-grade serous carcinomas, are believed to arise from the distal region of the Fallopian tubes [34]–[36]. Both the OSE and the Fallopian tubes are currently thought to be possible sources of neoplastic EOC and both are derived from the embryonic Müllerian duct [37]. CX_3_CL1 has been detected in the human endometrium [38] and Fallopian tubes [39]. We also detected CX_3_CL1 in the OSE, further indicating that the production of CX_3_CL1 by epithelial ovarian cells precedes tumorigenesis. CX_3_CL1 was also detected in benign and borderline tumor cells, suggesting that its production is not associated with malignancy.

Ovarian epithelial tumors are morphologically heterogeneous and are classified by pathologists into serous, clear cell, endometrioid and mucinous subtypes on the basis of histopathological examination. Each subtype is characterized by a specific mRNA profile, genetic risk factors and molecular features [34] [40]–[41], suggesting that ovarian carcinoma is a heterogeneous disease [42]. Despite this heterogeneity, we found no significant association between CX_3_CL1 levels and histological type in our series, which included representative specimens of all four principal histological types of EOC. Like GILZ and CXCL12, CX_3_CL1 is widely expressed in EOCs and its presence does not reflect the morphological heterogeneity of EOC [25] [28].

The chemokines, including CXCL12, are produced locally in ovarian tumors and contribute to tumor microenvironment [5]–[6]. Here, we identify CX_3_CL1 as another component of the EOC microenvironment. Epithelial cells from malignant ascites, tumor specimens and from three ovarian cancer cell lines, namely BG1, OVCAR3 and SKOV3, displayed staining for CX_3_CL1. CX_3_CL1 was confined to the cytoplasm and was absent from nuclei. The cells contained CX_3_CL1 with a molecular weight of 90 kDa corresponding to the membrane form of CX_3_CL1, from which the soluble form is derived by shedding [8]. The production of sCX_3_CL1 in culture supernatants paralleled that of the membrane-bound form, suggesting that the production of sCX_3_CL1 in EOC microenvironment was enhanced in tumors with strong CX_3_CL1 immunoreactivity. Interestingly, the local releasing of CX_3_CL1 may also depend on CXCL12, produced by epithelial ovarian malignant cells in EOC and known to regulate the cleavage of CX_3_CL1 from neurons [43]. We cannot exclude the possibility that CXCL12 stimulates the metalloproteinases involved in CX_3_CL1 cleavage in EOCs, as it does in neuronal cultures. Indeed, further investigation of this aspect is required to conclude.

The intensity of CX_3_CL1 staining and the fraction of tumor cells stained for CX_3_CL1 were variable in our cohort of 54 patients with advanced primary EOC. This heterogeneity in the production of CX_3_CL1 was positively correlated with GILZ levels. It does not exclude that certain EOC specimens produce CX_3_CL1 in the absence of GILZ, as shown in Table 3. That is also the case for cells from OSE and benign tumors, which never produce GILZ [25]. We then found that GILZ increased the production of CX_3_CL1 transcripts and proteins, consistent with a transcriptional regulation of *CX_3_CL1* by GILZ in malignant tumor cells. GILZ interferes with various transcription factors [44]–[45] or may directly control the transcriptional activity of proteins [46]. Further investigation is thus required to determine whether GILZ promotes *CX_3_CL1* transcription, as do several oncogenes including Ras, Myc and oncosuppressor genes, such as mutant p53, for chemokines and their receptors (reported in [47]).

GILZ is itself a proliferative factor in EOC [25], consistent with the correlation between CX_3_CL1 and Ki-67 levels in EOC specimens being a consequence of the upregulation of CX_3_CL1 production by GILZ. However, proliferative effects of CX_3_CL1 have been reported in smooth muscle cells [48]–[49] and we could not exclude the possibility that CX_3_CL1 may affect the rate of proliferation through autocrine action. In line with this hypothesis, we showed that the constitutive production of CX_3_CL1 by malignant epithelial ovarian cells led to the release of the soluble form of this chemokine, which binds to CX_3_CR1 present on tumor cells. Further *in vitro* experiments showed that CX_3_CL1 promoted BG1 cell proliferation through its binding to CX_3_CR1 as well as AKT activation, as previously reported for human prostate cancer cells [17]. The PI3K/AKT pathway transmits mitogenic signals and controls cell cycle progression in ovarian cancer [31] [33]. We previously reported that GILZ activates AKT in EOC [25]. Here AKT activation was clearly associated with the action of CX_3_CL1. Finally, our data are consistent with a model in which GILZ activates CX_3_CL1 and the chemokine acts alone to support ovarian tumor cell proliferation via CX_3_CR1. Thus, the activation by GILZ of CX_3_CL1 production may contribute to the proliferative effect of GILZ. It does not exclude that GILZ in itself has a proliferative action in EOC and activates AKT. To conclude, further studies are still needed to precise the molecular and cellular mechanisms underlying the respective contribution of GILZ and CX_3_CL1 to the proliferation of malignant cells in EOC. Cancer cells frequently grow more rapidly and have higher proliferation rates than normal cells. CX_3_CL1 may participate to this action in EOC through autocrine effects that should contribute to its pro-tumor potential. However, we can no longer exclude that CX_3_CL1 also contributes to other aspects of tumor biology, including immune cell recruitment and the anti-tumor response. That probably should make the prognostic value of this chemokine difficult to evaluate.

We show here that CX_3_CL1 is a component of the EOC microenvironment. Together with GILZ, this chemokine emerges as a regulator of cell proliferation in EOC. These results provide an encouraging starting point for elucidation of the functional importance of CX_3_CL1 in the progression of ovarian cancer and of the link between CX_3_CL1, GILZ and EOC proliferation demonstrated for the first time in this report. Few prognostic factors capable of accounting for tumor biology and disease course have been identified in ovarian cancer. The identification of molecular targets closely associated with cell proliferation might facilitate the development of personalized treatment and are of potential clinical relevance for the selection of the most appropriate treatment for certain cancer patients.

## Materials and Methods

### Ethics Statement

Formalin-fixed and paraffin-embedded tumors from primary surgery were recovered from healthy ovaries (most often the contralateral healthy ovary to the malignant one), benign tumors (serous and mucinous), borderline tumors (serous and mucinous), and granulosa tumors were provided by archival materials from patients treated at Antoine-Béclère Hospital (Service d'Anatomie et de Cytologie Pathologiques, Clamart, France) between 1998 and 2007. Approval was obtained from the ethics committee, Comité de Protection des Personnes, Ile de France, President, Pr Philippe Casassus, for all analyses of tumor material from clinical samples and archived material from patients diagnosed with an ovarian tumor (benign, borderline or malignant invasive). The study was carried out in accordance with good clinical practice guidelines, national laws, and the Declaration of Helsinki. All patients provided written informed consent.

Xenograft studies were carried out in strict accordance with the recommendations in the Guide for the Care and the Use of Laboratory Animal of the National Institutes of Health. The protocol obtained approval of ethics committee 26 for animal experimentation, Institut Gustave-Roussy at Villejuif, France. Agreement no. C92-023-0 for animal care, handling and experimentation is in accordance with European Union and French guidelines for the use of laboratory animals.

### Tissue samples

Immunohistochemical staining was carried out for CX_3_CL1, CXCL12, GILZ and Ki-67 in tissue specimens from primary invasive ovarian carcinomas taken for routine diagnosis and treatment purposes, from 54 patients treated surgically for ovarian cancer diagnosed at Antoine-Béclère Hospital between 1998 and 2007. The clinical and pathological characteristics of the patients are described (Table S1). None of the patients had received neo-adjuvant chemotherapy before surgery. Clinical stage was determined according to the International Federation of Gynecology and Obstetrics staging system (FIGO). Histological subtypes and grades were determined according to the criteria of the World Health Organization (WHO) classification [50].

### Immunostaining grading and score

Immunohistochemical staining for CX_3_CL1, GILZ, CXCL12 and Ki-67 was performed on 5 µm sections from paraffin-embedded tissues from healthy ovaries, benign, borderline and malignant epithelial ovarian tumors, granulosa tumors and xenograft samples. The paraffin was removed by incubation in xylene and the sections were rehydrated in a graded series of ethanol solutions and washed in 1X phosphate-buffered saline (PBS). Antigens were unmasked by incubation in 10 mmol/l sodium citrate buffer (Dako, Trappes, France) and heating to 90°C in a microwave oven. Sections were then incubated for 2 h at room temperature with the appropriate primary antibody (Ab), under the conditions detailed in Table S2. The sections were washed and incubated with a biotinylated secondary Ab for 1 h at room temperature, and then with streptavidin-horseradish peroxidase (-HRP) complex (LSAB kit, Dako). Sections were then counterstained with hematoxylin. Negative controls were carried out by applying the same procedure with omission of the primary Ab. Images were obtained on a Leica DMLB microscope equipped with standard optic objectives, at the indicated magnification, and were digitized directly with a Sony 3CCD color video camera.

Immunochemical staining was interpreted simultaneously by two independent investigators (FG and SP) blinded to the characteristics of the patients and clinical and pathological outcome. Immunostaining for CX_3_CL1, CXCL12, GILZ and Ki-67 was scored on the following scale (with a maximum score of seven): negative (0), 1 (weak intensity), 2 (moderate intensity) or 3 (strong intensity) combined with the percentage of positive cells scored as 0 (0%), 1 (1–10%), 2 (10–50%), 3 (50–80%), 4 (>80%), as recently reported [25] [28].

### Tumor cell enrichment from ascites

Tumor cell enrichment from ascites was based on the expression of CD326, a human epithelial antigen also known as EpCAM, one of the most frequently identified and highly expressed biomarkers in EOC [51]. CD326^+^ cells were positively selected on autoMACs columns (Miltenyi Biotec, Paris, France) from ascites samples collected with institutional review board (Antoine-Béclère Hospital) approval from a patient diagnosed with invasive EOC with peritoneal extension, as previously described [25]. The percentage of CD326^+^ cells in the positive fraction exceeded 80%, as shown by flow cytometry (FACSCalibur, BD Biosciences, France) with a PE-conjugated anti-human CD326 monoclonal Ab (mAb) (clone HEA 125, IgG1, Miltenyi Biotec).

### Cell lines

The human epithelial ovarian carcinoma cell line BG1, which was derived from a stage III solid tumor tissue from a patient (kindly provided by Dr G. Lazennec, INSERM U844, Montpellier, France), was maintained in Dulbecco's modified Eagle medium (DMEM) supplemented with 10% fetal bovine serum (FBS), 2 mmol/l L-glutamine and 0.1 mg/ml streptomycin. BG-1 clones stably overexpressing GILZ (pGILZ) or transfected with empty vector (CTRL) were generated as previously described [25]. The SKOV3 and OVCAR3 cell lines were purchased from the American Type Culture Collection (ATCC, Manassas, VA) and maintained in RPMI-1640 medium supplemented with 0.1 mg/ml streptomycin, 100 U/ml penicillin, 2 mmol/l L-glutamine and 10% FBS. The HEK 293T (ATCC) cell line was maintained in DMEM medium supplemented with 0.1 mg/ml streptomycin, 100 U/ml penicillin, 4 mmol/l L-glutamine and 10% FBS (Fisher Bioblock, Illkirch, France). All cell lines were maintained at 37°C, under an atmosphere containing 5% CO_2_.

### RT-PCR analyses

Total RNA was extracted from cultured cells, freshly frozen ovarian tissue samples and tumor samples harvested from mice, with the RNeasy Mini kit (Qiagen, Courtaboeuf, France), according to the manufacturer's instructions. The RNA was reverse transcribed to generate cDNA with random hexamers (Roche Diagnostics, Meylan, France) and Moloney murine leukemia virus reverse transcriptase (Fisher Bioblock). We amplified the resulting cDNA (1 µg) by conventional or real-time PCR on a Light Cycler instrument (Roche Diagnostics), with the FastStart DNA Master SYBER Green kit (Roche Diagnostics), and carried out quantification by the standard curve method. The primer sequences, predicted amplicon size and annealing temperature are shown in Table S3.

### [^3^H] thymidine uptake

Cells were used to seed 96-well plates, in triplicate, at a density of 1×10^4^ cells/well. They were grown to 60% confluence in DMEM medium supplemented with 10% FBS, for 24 h. The cells were then washed with PBS and cultured in charcoal-treated medium supplemented with 0.1% FBS. [^3^H] thymidine (0.5 µCi/well) (MP Biomedicals Europe, Illkirch, France) was added and the cells were incubated overnight. The amount of incorporated radioactivity was determined as previously described [52] and the results are expressed as counts per minute (cpm). The binding of CX_3_CL1 to CX_3_CR1 was antagonized using a modified CX_3_CL1 analog prepared from E. Coli inclusion bodies using standard procedures as recently described [29].

### Western blotting

Cells (2×10^6^) were lysed as previously described [52]. Equivalent amounts of protein were separated by SDS-polyacrylamide gel electrophoresis (SDS-PAGE) and transferred to nitrocellulose membranes (Hybond-ECL, GE Healthcare, Orsay, France). Nonspecific binding was prevented by incubating the membranes with blocking buffer (50 mM Tris-HCl, 150 mM NaCl, 0.1% TWEEN-20 and 5% skim milk powder) for 1 h at room temperature. The membranes were then incubated overnight at 4°C with specific primary Abs (Table S2). These Abs were then detected by incubating the membrane with an HRP-conjugated secondary Ab (GE Healthcare) for 1 h at room temperature. The membrane was placed against film (GE Healthcare) and the bands were visualized by enhanced chemiluminescence (Perkin Elmer, Courtaboeuf, France). ScanAnalysis software (Biosoft, Cambridge, United Kingdom) was used for densitometric analysis. All bands were normalized with respect to ß-actin.

### Flow cytometry analysis

CX_3_CR1 was detected with or without the addition of acid buffer (50 mM glycine, 120 mM NaCl, pH = 2.7−3) and incubation for 3 minutes at 4°C to remove surface-bound ligands from the receptor. The cells were washed with cold PBS supplemented with 2% FBS and incubated for 20 minutes on ice with 10 µg/ml FITC-conjugated anti-hCX_3_CR1 mAb (Table S2). For the detection of the cytoplasmic pool of CX_3_CR1, cells were permeabilized with BD Cytofix/Cytoperm™ reagent (BD Pharmingen, Le Pont De Claix, France) according to manufacturer's instructions, before labeling. At least 10,000 events were acquired for each sample. FITC-conjugated IgG2b was used as a negative isotype control (Clinisciences, Montrouge, France). Data were acquired with a FACSCalibur flow cytometer and analyzed using the CellQuest software (BD Biosciences).

### ELISA

Soluble CX_3_CL1 was detected with the human CX_3_CL1/Fractalkine Quantikine ELISA kit (R&D Systems, Lille, France), according to the manufacturer's instructions. Absorbance was read at 450 nm and sCX_3_CL1 concentration was extrapolated from the standard concentration curve. The minimum detectable concentration was 0.018 ng/ml.

### *In vivo* xenografted tumor model

Male nude athymic mice (Harlan, Gannat, France), purchased at four weeks of age, were used for xenograft studies. CTRL or pGILZ BG1 cells (8×10^6^ cells in 200 µl PBS) were injected subcutaneously into the flanks of separate five-week-old mice. Tumor size was determined with calipers, every five days. All mice were killed humanely after 35 days. Tumor volume was calculated as follows: (L x W^2^) x 0.5, (L: length; W: width).

### Statistical analysis

Statistical analyses were performed with StatEL statistical software (Adscience, Paris, France). Spearman's test, univariate analysis, was used to assess the correlations between CX_3_CL1, GILZ and Ki-67 levels. Differences between groups were assessed with the Welch two-sample unpaired *t* test, and by two-tailed paired *t* tests. Fisher's exact tests were used to assess the significance of differences between clusters. Clustering, a widely used approach for subtype identification, was carried out with the hierarchical agglomerate clustering approach, in StatEL software, with Pearson's correlation function for quantitative data.

## Supporting Information

Table S1 **Clinical and histological parameters of patients.**
(DOC)

Table S2 **Antibodies used for immnohistochemistry, western blotting and flow cytometry.**
(DOC)

Table S3 **Primer sequences used for conventional and real-time PCR.**
(DOC)
