# Supplementary material for: Identification of the Chemokine CX3CL1 as a New Regulator of Malignant Cell Proliferation in Epithelial Ovarian Cancer
Source: PLoS One. 2011 Jul 7;6(7):e21546. doi: 10.1371/journal.pone.0021546 (PMC3131275; doi:10.1371/journal.pone.0021546)
Supplement: Table S1 — Clinical and histological parameters of patients. (DOC) [file pone.0021546.s001.doc]

**Supplementary Table 1**. Clinical and histological parameters of patients

Patient number Percentage Median Min-Max

***Clinical parameters***

Invasive ovarian tumors 54

Age (years) 56 [30-86]

***Histological type***

Serous 30 55.6

Endometrioid 7 13

Mucinous 8 14.8

Clear-cell 6 11.1

Undifferentiated 3 5.5

***Disease stages***

FIGO stages IA-IC 15 (12 IA + 3 IC) 27.8

FIGO stages IIA-IIC 4 (2 IIA + 2 IIC) 7.4

FIGO stages IIIA-IIIC 33 (2 IIIA + 6 IIIB + 25 IIIC) 61.1

FIGO stage IV 2 IV 3.7

***Histological grade***

Grade 1 8 14.8

Grade 2 13 24.1

Grade 3 24 44.4

Undetermined 9 16.7
