# Supplementary material for: Identification of the Chemokine CX3CL1 as a New Regulator of Malignant Cell Proliferation in Epithelial Ovarian Cancer
Source: PLoS One. 2011 Jul 7;6(7):e21546. doi: 10.1371/journal.pone.0021546 (PMC3131275; doi:10.1371/journal.pone.0021546)
Supplement: Table S2 — Antibodies used for immnohistochemistry, western blotting and flow cytometry. (DOC) [file pone.0021546.s002.doc]

**Supplementary Table 2.** Antibodies used for immnohistochemistry, western blotting and flow cytometry

| Protein | **Experimental**  **procedure** | Species | Type/Clone | **Dilution** | Origin |
| --- | --- | --- | --- | --- | --- |
| **CX_3_CL1** | IHC/WB | Goat | Polyclonal | 1:20/1:100 | R&D Systems |
|  |  |  |  |  |  |
| **GILZ** | IHC/WB | Rabbit | Polyclonal | 1:100/1:300 | Tébu |
| **CXCL12** | IHC | Mouse | K15C | 1:100 | Pasteur Institute  (U819 Inserm) |
| **pAKT (Ser 473)** | WB | Rabbit | Polyclonal | 1:1000 | Cell signaling |
| **Ki-67** | IHC | Mouse | MIB-1 | 1:50 | Dako |
| **AKT** | WB | Rabbit | Polyclonal | 1:1000 | Cell signaling |
| **β-actin** | WB | Goat | Polyclonal | 1:1000 | Tébu |
| **CX_3_CR1-FITC** | FC | Rat | 2A9-1 | 1:10 | CliniSciences |
| **CD45-PerCP** | FC | Mouse | HI30 | 1:10 | Biolegend |

WB, Western blot

IHC, Immunohistochemistry

FC, Flow cytometry
