# Supplementary material for: Identification of the Chemokine CX3CL1 as a New Regulator of Malignant Cell Proliferation in Epithelial Ovarian Cancer
Source: PLoS One. 2011 Jul 7;6(7):e21546. doi: 10.1371/journal.pone.0021546 (PMC3131275; doi:10.1371/journal.pone.0021546)
Supplement: Table S3 — Primer sequences used for conventional and real-time PCR. (DOC) [file pone.0021546.s003.doc]

**Supplementary Table 3.** Primer sequences used for conventional and real-time PCR

| **Target** | **PCR procedure** | **Forward and reverse primer sequences**  (Eurogentec, France) | **Size of**  **amplicons** | **Annealing temperature** |
| --- | --- | --- | --- | --- |
| ***CX_3_CL1*** | Conventional | 5’-CCGAAGGAGAGCAATGGGTCAA-3’  5’-TTCCTACTCCCCCTGCTCAT-3’ | 387 bp | 57°C |
| ***CX_3_CL1*** | Real-time | 5’-GCTTTGCTCATCCACTATCAACA-3’  5’-GCTCCAGGCTACTGCTTTCG-3’ | 282 bp | 60°C |
| ***CX_3_CR1*** | Conventional | 5’-TTCCTACTCCCCCTGCTCAT-3’  5’-CCTGTATGGGAAATGCCTGG-3’ | 340 bp | 62°C |
| ***GILZ*** | *Conventional /  Real-time | 5’-TCTGCTTGGAGGGGATGTGG-3’  5’-ACTTGTGGGGATTCGGGAGC-3’ | 294 bp | 62 / 65°C |
| ***β-actin*** | *Conventional /  Real-time | 5’-TTCCTACTCCCCCTGCTCAT-3’  5’-CCTGTATGGGAAATGCCTGG-3’ | 237 bp | 55 / 60°C |

* the same primers were used for both conventional and real-time PCR.
